# Analysis of Cellular Activity and Induction of Inflammation in Response to Short-Term Exposure to Cobalt and Chromium Ions in Mature Human Osteoblasts

**DOI:** 10.3390/ma12172771

**Published:** 2019-08-28

**Authors:** Anika Jonitz-Heincke, Marie-Luise Sellin, Anika Seyfarth, Kirsten Peters, Brigitte Mueller-Hilke, Tomas Fiedler, Rainer Bader, Annett Klinder

**Affiliations:** 1 Biomechanics and Implant Technology Research Laboratory, Department of Orthopedics, Rostock University Medical Centre, Doberaner Strasse 142, 18057 Rostock, Germany; 2 Department of Cell Biology, Rostock University Medical Center, Schillingallee 69, 18057 Rostock, Germany; 3 Institute for Immunology, Rostock University Medical Center, Schillingallee 70, 18057 Rostock, Germany; 4 Institute for Medical Microbiology, Virology and Hygiene, Rostock University Medical Center, Schillingallee 70, 18057 Rostock, Germany

**Keywords:** osteoblasts, corrosion, ions, particles, osteolysis, inflammation

## Abstract

In aseptic loosening of endoprosthetic implants, metal particles, as well as their corrosion products, have been shown to elicit a biological response. Due to different metal alloy components, the response may vary depending on the nature of the released corrosion product. Our study aimed to compare the biological effects of different ions released from metal alloys. In order to mimic the corrosion products, different metal salts (CoCl_2_, NiCl_2_ and CrCl_3_ × 6H_2_O) were dissolved and allowed to equilibrate. Human osteoblasts were incubated with concentrations of 10 µM to 500 µM metal salt solutions under cell culture conditions, whereas untreated cells served as negative controls. Cells exposed to CoCr28Mo6 particles served as positive controls. The cell activity and expression of osteogenic differentiation and pro-osteolytic mediators were determined. Osteoblastic activity revealed concentration- and material-dependent influences. Collagen 1 synthesis was reduced after treatment with Co(2+) and Ni(2+). Additionally, exposure to these ions (500 µM) resulted in significantly reduced OPG protein synthesis, whereas RANKL as well as IL-6 and IL-8 secretion were increased. *TLR4* mRNA was significantly induced by Co(2+) and CoCr28Mo6 particles. The results demonstrate the pro-osteolytic capacity of metal ions in osteoblasts. Compared to CoCr28Mo6 particles, the results indicated that metal ions intervene much earlier in inflammatory processes.

## 1. Introduction

The wear and corrosion of metal implant materials are the main risk factors for aseptic loosening and implant failure in orthopedic surgery. Although metal surfaces are protected by a passive oxygen layer, electrochemical reactions are still present at the implant site [1]. Different components can cause galvanic effects resulting in oxidation and reduction reactions on the metal surfaces. As a consequence, a continuous exchange of electrons as well as ions between the metal and surrounding liquid is maintained [2]. Head-neck taper corrosion is the most prominent example of this in orthopedic surgery, especially for large-diameter metal-on-metal bearings [3], but also for metal-polyethylene bearings [4]. Another type of corrosion is the intracellular reduction of wear particles in cells, especially in macrophages. When metal particles are taken up via active mechanisms or internalization, the degradation of these nanostructures is promoted by the lysosomal environment [5]. Due to the acidic pH in the cellular components, the release of metal ions in different oxidation states is fostered [6]. Besides the intracellular accumulation of these toxic substances, metal ions are released into the extracellular environment where other cell types can be directly influenced. However, osteolysis is not directly induced by the release of ions but rather the reaction of ions with biomolecules, which leads to higher bioactivity and destruction [7]. A third mechanism of corrosion has been described as direct inflammatory cell-induced corrosion, which occurs on retrieved metal implants. Here, the direct cell attack and concomitant formation of a ruffled cell membrane have been assumed as a trigger for corrosive processes [8].

Apart from titanium-aluminum-vanadium, cobalt-chromium-molybdenum (CoCr28Mo6) is the most prominent alloy in orthopedic surgery. While cobalt alloys are characterized by their high strength, fracture toughness, and excellent biocompatibility, the degradation of the surface due to corrosion, or a combination of corrosion and wear, can lead to harmful local and systemic side-effects in the human body [2,6,9]. Cytotoxic effects of Co, Cr, Ti, and nickel (Ni), which present themselves mainly as apoptosis, necrosis, and inhibitory effects on the DNA repair mechanism, are caused by means of chromosomal damage and oxidative stress [2,10]. 

Despite these similar outcomes, different metal ions vary with regard to local and systemic effects. Cr ions are able to react with phosphate compounds to form stable chromium phosphates (CrPo_4_) which then accumulate in the peri-implant tissue [2,6]. In contrast, Co ions can circulate within the body, affecting many organs and causing, e.g., neurological, cardiological, or endocrine symptoms [2]. The varying behavior is also reflected in the different concentrations found locally as well as in the blood of patients suffering from aseptic loosening and metallosis. As blood is easily accessible, there is a sound base of data. Serum cobalt concentrations of 2–7 µg/L (0.002–0.007 ppm) or 10 µg/L (0.010 ppm) were stated respectively in a European multidisciplinary consensus statement [11] or by the Mayo Clinic [12] as indicative of metallic wear and implant loosening. The systemic concentrations reported in patients with acute cobalt poisoning after excessive wear particle release with fatal or near fatal outcomes were approximately a hundred times higher (serum levels of around 0.40–0.64 ppm cobalt and 0.05–0.08 ppm chromium) than the suggested threshold, with even higher values excreted in urine [13,14,15,16,17]. While cobalt concentrations are higher than chromium concentrations in serum this is reversed in the periprosthetic tissue, partially due to the above mentioned reasons. Scharf et al. [6] measured an average of 0.17 ppm for cobalt and 1.60 ppm for chromium in tissue surrounding of total hip implants with metal on metal (MoM) bearings in nine patients with revisions due to adverse local tissue reactions [6]. In a more recent publication by Kuba et al. (2019) revision patients with long-term surviving implants displayed mean values of 6.52 ± 16.38 ppm for cobalt and 8.88 ± 26.88 ppm for chromium in periprosthetic tissue—concentrations in joint fluid were around 0.048 ppm for Co and 0.167 ppm for Cr [18]. However, in the rare cases, where wear and loosening led to acute poisoning, local concentrations of 41 ppm Co [16] up to 397.8 ppm Co and 236.0 ppm Cr [19] in periprosthetic tissue, or 76 ppm Co and 126.5 ppm Cr in locally aspirated liquid [14], were reported. These high local concentrations induce detrimental effects and an inflammatory response to metal ion intoxication can be locally found in the periprosthetic tissue. Tissue necrosis is associated with macrophage and lymphocyte infiltration [6] and the secretion of a variety of pro-inflammatory mediators (e.g., interleukins (IL), tumor necrosis factor (TNF)), especially from macrophages, is induced [7]. However, various studies have revealed that bone forming osteoblasts are also affected by metal ions, resulting in reduced proliferation and differentiation capacity as well as inducing pro-inflammatory cytokine release [2,20,21]. In this context, it is interesting that mineralized bone tissue seems to be prone to accumulate metal debris as the concentrations of 38–413 ppm Co here were considerably higher than those reported above for soft tissue [22].

In a previous in vitro study, we exposed human osteoblasts and macrophages to a mixture of Co and Cr ions (200 µg/L or 0.2 ppm) and analyzed their pro-osteolytic capacity in the cell cultures [21]. Although we observed marginal detrimental effects in single cell cultures, both a clear impact on osteoblastic function and the initiation of pro-osteolytic events were detectable under co-culture conditions of human osteoblasts and macrophages. However, a limitation of this study was the missing knowledge of the proportion of either Co or Cr ions. As a result, proven effects could not be attributed to one metal ion species or another. Thus, for further understanding of differential effects of Co and Cr ions, we now exposed human osteoblasts to different concentrations of Co and Cr metal salts. Based on previous studies by Scharf et al. (2014) and Drynda et al. (2018) we used ion concentrations between 10 µM and 500 µM [6,23]. These concentrations correspond to 0.59–29.47 ppm Co and 0.52–26.00 ppm Cr and are similar to those reported in periprosthetic tissue by Kuba et al. (2019) [18], but lower than those in bone tissue [22]. However, due to the analytical methodology it is difficult to specify whether the determined metal concentration originated from metal particles or metal ions.

Additionally to Co and Cr, nickel ions—which are known to have a higher toxicity on osteoblastic viability and proliferation than Co and Cr [2,24]—were investigated since forced corrosion of CoCr28Mo6 particles resulted in considerable levels of Ni ions in the solution that exceeded the amount of molybdenum [21]. Simultaneously, human osteoblasts were also treated with CoCr28Mo6 particles in order to clarify whether corrosion products exhibit similar or differing effects to abrasion particles in cells. Hence, the objective of this study was to assess the effects of relevant corrosion products on cell survival, bone remodeling, and the release of pro-osteolytic mediators in mature human osteoblasts. Therefore, short-term studies with different metal ion concentrations were carried out to determine the threshold at which metal salts are harmful for cell survival.

## 2. Materials and Methods

### 2.1. Preparation of Metal Salt Solutions

The following metal salts were purchased from Sigma-Aldrich (Sigma-Aldrich Chemie GmbH, Munich, Germany): Cobalt(II) chloride (purum p.a., anhydrous, purity ≥ 98.0% (KT)), Nickel(II) chloride (anhydrous, powder, purity 99.99% trace metals basis) and Chromium(III) chloride hexahydrate (purum p.a., purity ≥ 98.0% (RT)). Stock solutions of a concentration of 100 mM were produced by dissolving the appropriate amount of salt in Aqua ad iniectabilia (B. Braun Melsungen AG, Melsungen, Germany). Stock solutions were stored in Schott Duran^®^ laboratory glass bottles (Schott AG, Mainz, Germany) at 4 °C in the dark. Before the use in the experiments the chromium salt stock solution was equilibrated according to Drynda et al. (2018) for at least two weeks until the color of the solution changed completely from emerald green to purple, indicating the presence of the stable species of [Cr_3_ + (H_2_O)_6_]Cl_3_ [23]. For cell experiments, the stock solutions were further diluted with cell culture media to expose cells to concentrations of 10 µM, 50 µM, 100 µM and 500 µM, respectively. 

### 2.2. Isolation and Cultivation of Human Primary Osteoblasts

Isolation of human primary osteoblasts (n = 11; eight female donors: mean age 70 years ± 18 years; three male donors: mean age 70 years ± 13 years) was performed according to the protocol of Lochner et al. (2011) [25]. The bone marrow was extracted under sterile conditions from femoral heads of patients undergoing primary hip replacements who had given written consent (Local Ethical Committee AZ: 2010-10). Extracted spongiosa was washed three times in phosphate-buffered saline (PBS, Biochrom AG, Berlin, Germany) followed by an enzymatic digestion with collagenase A and dispase II (both from Roche, Penzberg, Germany) at 37 °C. The material was filtered through a cell strainer (70 µm pores, BD Biosciences, Bedford, UK) and the isolated cell suspension was centrifuged at 118× g for 10 min. The cell sediment was re-suspended in cell culture medium. Human primary osteoblasts were cultivated in a special formulation of calcium-depleted Dulbecco’s modified Eagle medium (DMEM, Pan Biotech GmbH, Aidenbach, Germany) containing 10% fetal calf serum (Pan Biotech GmbH), 1% penicillin/streptomycin, 1% amphotericin b, 1% HEPES buffer, and the osteogenic additives L-ascorbate-2-phosphate (50 µg/mL), β-glycerophosphate (10 mM), as well as dexamethasone (100 nM) (all: Sigma-Aldrich, Munich, Germany) at 37 °C and 5% CO_2_. These specific conditions, in particular the addition of osteogenic factors dexamethasone, β-glycerophosphate and L-ascorbate-2-phosphate, led to the differentiation of the isolated pre-osteoblasts into mature osteoblasts in standard cell culture flasks (polystyrene) [26,27]. The osteoblastic phenotype was analyzed by alkaline phosphatase staining (with fuchsin+substrate-chromogen; DAKO, Hamburg, Germany). Cells from passage 3 were harvested for subsequent cell culture experiments as follows. Cells were washed with PBS, trypsinized, and centrifuged at 118× *g*. If not otherwise stated, 30,000 cells (in duplicate) were transferred into a well of a 24-well cell culture plate allowing cell adherence over 24 h at 37 °C and 5% CO_2_. Afterwards, cells were exposed to different concentrations of metal salts. Untreated cells served as negative controls whereas osteoblasts treated with CoCr28Mo6 particles (particle concentration: 0.01 mg/mL) [28,29] were used as positive control. For the comparison of the influence of different culture media, matured cells from passage 3 were not only seeded in the above described Ca-depleted osteogenic medium but also in standard Dulbecco’s modified Eagle medium (Gibco™ DMEM Glutamax, ThermoFisher Scientific, Waltham, MA, USA) with osteogenic additives L-ascorbate-2-phosphate (50 µg/mL), β-glycerophosphate (10 mM) and dexamethasone (100 nM) (all: Sigma-Aldrich) before treatment.

### 2.3. Cellular Activity

The cell activity of ion-exposed human osteoblasts was determined after 48 h of exposure. Osteoblasts (10,000 cells per well, in duplicates) were seeded in black 96-well cell culture plates (Thermo Fisher Scientific Inc., Waltham, MA, USA). After 24 h of adherence under standard cell culture conditions, cells were treated with metal salts or particles. Supernatants were removed after 48 h and the water soluble tetrazolium salt (WST-1) assay (Roche, Penzberg, Germany) was performed according to the manufacturer’s recommendations. Cells were incubated with a defined volume of WST-1/medium (1:10 ratio) reagent for 30 min. Subsequently, supernatants were transferred in a 96-well cell culture plate and absorbance at 450 nm (reference wave length: 630 nm) was determined in a Tecan Infinite^®^ 200 Pro microplate reader (Tecan Group AG, Maennedorf, Switzerland). Afterwards, the same cells were used to quantify cell numbers using the CyQUANT^®^ NF Cell Proliferation Assay Kit (Invitrogen, Thermo Fisher Scientific Inc., Waltham, MA, USA) according to the recommendations of the manufacturer. This combined approach was possible as the CyQUANT^®^ kit measures the emission of fluorescence at 520 nm after the lysis of the cells and the subsequent binding of a proprietary green fluorescent dye, CyQUANT^®^ GR dye, to cellular nucleic acids. The fluorescence signal depends solely on the amount of DNA present in the sample. In order to relate the fluorescence signal to an actual cell number, a cell number calibration curve was prepared with defined cell numbers between 0 and 20,000 cells in duplicate prior to each experiment. The calibration curve was then used to determine the cell number of treated cells per well. Fluorescence intensity was measured with the Tecan Infinite^®^ 200 Pro (Tecan Group AG, Maennedorf, Switzerland) microplate reader (excitation: 485 nm, emission: 535 nm). Cellular activity was calculated by dividing WST-1 results by the respective cell number as measured by CyQuant (see Supplementary Materials Figures S1 and S2). Since the activity values per cell were very small, results are presented as activity per one million cells for easier illustration of data.

Additionally, microscopic examinations of cell cultures were carried out after 48 h of incubation. Morphology of human osteoblasts was documented via light microscopy using a 200× magnification (Nikon ECLIPSE TS100, Nikon GmbH, Duesseldorf, Germany). The formation of actin filaments within cell structures was visualized by actin staining and DAPI counterstain following the procedure described by Klinder et al. (2018) [28]. Pictures for actin staining were taken with 400× magnification at 500 nm where actin stain fluoresced green. Cell nuclei were visualized with DAPI stain at 400 nm and showed a blue fluorescence. The respective pictures were taken from exactly the same spot and superimposed upon each other with the help of Adobe Photoshop CS6 image processing software Version 13.0.1 (Adobe Systems Software Ireland Ltd., Dublin, Ireland).

### 2.4. Gene Expression Analysis

RNA isolation was carried out using the peqGOLD Total RNA Kit (VWR International GmbH, Hanover, Germany) following the manufacturer’s protocol. RNA was eluted into a fresh sterile tube using RNase free water and RNA concentration was measured using the Tecan Infinite^®^ 200 (Tecan Group AG, Maennedorf, Switzerland) microplate reader and NanoQuant Plate™ with RNase free water as blank. The purity of the isolated RNA was assessed and median ratios at 260/280 nm of 2.10 (2.10–2.11), 2.10 (1.78–2.14), 2.13 (2.04–2.19), 2.11(1.90–2.13) and 2.09 (2.08–2.11) were recorded for untreated, Co-treated, Cr-treated, Ni-treated and particle-treated samples, respectively. After RNA isolation, a reverse transcriptase polymerase chain reaction (RT-PCR) was used to transcribe the RNA into cDNA. Here, the High Capacity cDNA Reverse Transcription Kit (Applied Biosystems, Foster City, CA, USA) was used. A master mix was prepared as described in the manufacturer’s protocol. The specific amount of each RNA sample containing 200 ng RNA was calculated using the results from concentration measurement and added up to 10 μL with RNase free water in PCR tubes. Subsequently, 10 μL of master mix was added and mixed well. The samples were placed in a thermocycler (Analytik Jena, Jena, Germany) and the following RT-PCR protocol was used: 10 min at 25 °C, 120 min at 37 °C, 15 s at 85 °C. Afterwards, samples were diluted in additional 20 μL RNase free water and stored at −20 °C.

To determine the expression level of differentiation- and inflammation-associated genes, the cDNA of treated and untreated cells was used to perform a semiquantitative real-time (q-PCR) with SybrGreen. For the PCR reaction, the 2× innuMIX qPCR MasterMix SyGreen (Analytik Jena, Jena, Germany) was used following the manufacturer’s protocol. To that master mix, 0.5 μL of forward and reverse primer (12 μM), respectively, as well as 3 μL of Aqua dest. were added. For each sample, 1 μL template cDNA (in duplicates) was pipetted onto the bottom of a 96-well PCR plate and filled up with 9 µL of the mentioned master mix. The used primer sequences of osteogenic (Col1A1, ALP) and pro-osteolytic mediators (IL-6, IL-8) are listed in Table 1. Distilled water, instead of cDNA, served as negative control. The plate was sealed with adhesive foil and placed in the qTower 2.0 (Analytik Jena, Jena, Germany). Gene expression analysis was done under the following conditions: initial activation time of 2 min at 95 °C, 40 times of rotation of denaturation for 5 s at 95 °C and annealing/elongation for 25 s at 60–65 °C. A cycle of threshold (Ct) of 28 was set as limit. The relative expression of each gene compared to the housekeeping gene hypoxanthine guanine phosphoribosyl transferase (HPRT) was calculated using the equation: ΔCt = Ct_target_ − Ct_HPRT_. The relative amount of target mRNA of cells treated with metal salts and controls was calculated using 2^(−ΔΔCt)^ with ΔΔCt_treatment_ = ΔCt_treated_ − ΔCt_control_.

### 2.5. Protein Analysis

The protein contents of bone remodeling markers (pro-collagen type 1 (C1CP), osteoprotegerin (OPG), receptor activator of nuclear factor κb ligand (RANKL)) and pro-inflammatory mediators (interleukin (IL) 6 and 8) were determined in the supernatant of control and ion-exposed osteoblasts. For this purpose, the supernatants were collected and stored at −20 °C prior to quantification. C1CP was determined using the C1CP ELISA (Quidel, Marburg, Germany) according to the manufacturer’s recommendations. Absorbance was measured at 405 nm (reference wave length: 630 nm) using the Tecan Infinite^®^ 200 Pro (Tecan Group AG, Maennedorf, Switzerland) microplate reader. A standard curve was prepared to calculate protein concentration in samples. OPG and RANKL were determined via LEGENDplex™ (BioLegend, San Diego, CA, USA) using fluorescence-labeled beads. These beads are conjugated with the specific antibody on its surface. The samples were incubated with the antibody-conjugated beads, thus forming capture bead-analyte-detection antibody sandwiches. Afterwards, streptavidin-phycoerythrin was added, which bound to the biotinylated detection antibodies, providing fluorescent signal intensities in proportion to the amount of bound analytes. Fluorescence intensities (excitation: 575 nm, emission: 660 nm) in samples were analyzed on a flow cytometer (FACSAria™ IIIu, BD Biosciences). The concentrations of OPG and RANKL were quantified using a standard curve generated in the same assay as well as LEGENDplex^TM^ Data Analysis Software v8 (BioLegend, San Diego, CA, USA).

Soluble proteins of IL6 and IL8 were quantified via eBioscience™ Human IL-6/IL-8 ELISA Ready-SET-Go!™ Kits (both: ThermoFisher Scientific, Waltham, MA, USA) according to the instructions of the manufacturer. Absorbance was measured at 405 nm (reference wave length: 630 nm) using the Tecan Infinite^®^ 200 Pro (Tecan Group AG, Maennedorf, Switzerland) microplate reader. Sample concentrations were calculated using a standard curve, respectively. 

Finally, all protein contents within the samples were normalized to the overall protein content which was quantified by the Qubit Protein Assay Kit and Qubit 1.0 (both: Invitrogen) according to the manufacturer’s instructions.

### 2.6. Data Analysis and Illustration

Data analysis and illustration were performed by GraphPadPRISM v.7.02 (GraphPad Inc., San Diego, CA, USA). Results are shown as box plots. Boxes depict interquartile ranges, horizontal lines within boxes depict medians, and whiskers depict maximum and minimum values. For cell culture experiments, human osteoblasts were used in duplicates with a minimum of four independent donors. While gene expression results are depicted as percentage of 2^(−ΔΔCt)^ for a better visualization of the changes with the untreated control (0 µM) set as 100%, the underlying statistical analysis was performed with the ΔCt values to allow the statistical comparison to the untreated control (0 µM). For the statistical analysis of protein data the values of the specific protein amount normalized to total protein content were used. 

Statistical comparisons regarding the influence metal salts at different concentrations on cellular activity, gene expression, and protein synthesis were performed with repeated measures (RM) two-way analysis of variance (ANOVA) with “type of metal salt” and “concentration” as variables. Tukey’s multiple comparisons test was used for post hoc testing. Results after particle exposure (positive control) were compared to untreated samples (negative control) by either paired t-test or Wilcoxon matched-pairs test depending on normal distribution of data according to Shapiro-Wilk testing. The effects of the different cell culture media were analyzed with RM two-way ANOVA with “type of media” and “concentration” as variables. Post hoc testing was performed with Bonferroni’s multiple comparison test. Significances were set to a p-value less than 0.05. Further details of statistical tests are indicated in the results section and the figure legends.

## 3. Results

### 3.1. Influence of Metal Ions on Cellular Activity

**Figure 1:**
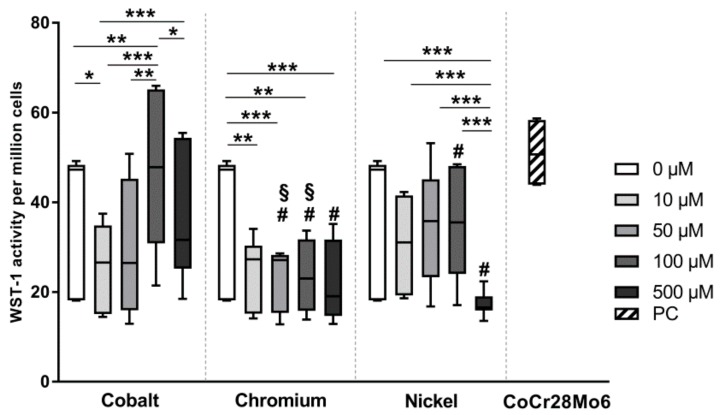
Cell activity of human osteoblasts after exposure to metal salts. Untreated cells served as controls (0 µM) while treatment with CoCr28Mo6 particles (0.01 mg/mL) was used as positive control (PC). Osteoblasts were treated with different concentrations of Co(2+), Cr(3+) and Ni(2+) over 48 h. Afterwards metabolic activity was determined via water soluble tetrazolium salt (WST-1) assay followed by cell number analysis using CyQUANT NF Cell Proliferation Assay. Data are shown as metabolic activity per million cells, depicted as box plots (n = 7). Significance was calculated with concentration-dependent differences: * *p* < 0.05, ** *p* < 0.01, *** *p* < 0.001; differences to cobalt: ^#^ *p* < 0.05; differences to nickel: ^§^ *p* < 0.05.

Evaluation of cell activity was tested via cell number determination and WST-1 assay (see Supplementary Materials Figures S1 and S2). In Figure 1 the metabolic activity of ion-exposed osteoblasts per million cells is depicted in comparison to negative (untreated) and positive (CoCr28Mo6 particles) controls. Cellular activity was influenced by the concentration (*p* = 0.0006) and the type of metal salt (*p* = 0.0002) with a strong interaction between both factors (*p* < 0.0001). Post hoc analysis showed that in comparison to untreated cells, the lowest concentration (10 µM) of Co ions led to decreased cell activity (*p* = 0.0430), while a concentration of 100 µM Co ions resulted in significantly enhanced cell activity levels compared to untreated control and the lower concentrations of 10 µM and 50 µM (*p* = 0.0048, *p* < 0.0001 and *p* < 0.0001, respectively). Moreover, exposure to 100 µM of Co ions and treatment with CoCr28Mo6 particles resulted in similar activity levels. However, when further increasing the concentration of cobalt salt to 500 µM, cellular activity decreased again compared to 100 µM (*p* = 0.0430). Metabolic activity of human osteoblasts exposed to Cr ions was significantly lower than untreated controls but this effect was not concentration-dependent (*p* = 0.0046, *p* = 0.0007, *p* = 0.0023 and *p* = 0.0004 of 10, 50, 100 and 500 µM all compared to untreated control, respectively). Exposure to the highest Ni(2+) concentration (500 µM) led to significantly reduced metabolic activity compared to all lower concentrations of Ni salt, as well as to untreated cells (*p* < 0.0001, *p* = 0.0005, *p* < 0.0001 and *p* < 0.0001 of 0, 10, 50 and 100 µM all compared to 500 µM, respectively). 

When comparing the different metal salts, exposure to Cr(3+) (50 µM, 100 µM and 500 µM) resulted in significantly reduced activity levels compared to Co ions (*p* = 0.0156, *p* < 0.0001 and *p* < 0.0001, respectively). For Ni ions there were still significantly decreased metabolism rates for concentrations 100 μM and 500 μM detected when compared to Co (*p* < 0.001 and *p* < 0.0001 at 100 µM and 500 µM, respectively). 

**Figure 2:**
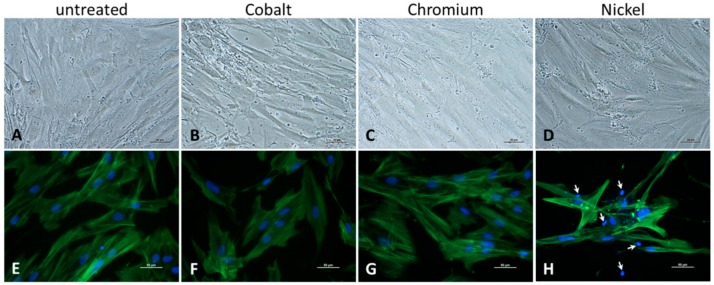
Determination of cell morphology of ion-exposed human osteoblasts (500 µM) after 48 h of incubation. (**A**–**D**): Phase contrast microscopy (scale bar: 20 µm); (**E**–**H**): Fluorescence staining of actin filaments and cell nuclei (scale bar: 50 µm).

The evaluation of cell morphology after exposure to metal salts was carried out with light microscopy and actin staining. Light microscopy revealed a tendency to morphological changes after treatment with Co and Ni ions (Figure 2B,D). Compared to untreated and Cr(3+)-exposed osteoblasts, cells seemed to be more fusiform without clearly formed filopodia for cell connections. Additionally, the actin stain of cells revealed a decrease in cell number after treatment with the bivalent ions Co(2+) and Ni(2+). While actin filaments were clearly visible in the control (Figure 2E) and Cr(3+)-exposed cells (Figure 2G), a weakening of the fluorescence signal was observed in the Co(2+) group (Figure 2F). The treatment with Ni ions not only led to a reduction in cell number (Figure S1) but also seemed to affect the cytoskeleton of the cells. Partly, cells were completely negative for actin fluorescence staining with only the counterstained nucleus visible (indicated by arrows in Figure 2H) or the cells were only stained along the cell membrane with no visible intracellular network structure.

### 3.2. Influence of Cell Culture Medium on Cell Activity after Exposure to Metal Salts

**Figure 3:**
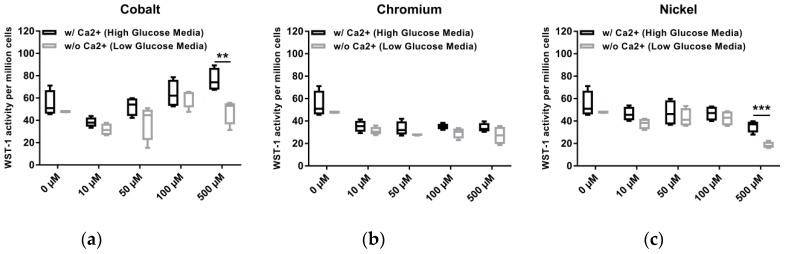
Comparison of cell activity of human osteoblasts after exposure to metal salts in Ca(2+) containing (high glucose medium) and Ca(2+) depleted (low glucose medium). Untreated cells served as negative control (0 µM). Osteoblasts were treated with different concentrations of (**a**) Co(2+), (**b**) Cr(3+) and (**c**) Ni(2+) over 48 h. Afterwards metabolic activity was determined via water soluble tetrazolium salt (WST-1) assay followed by cell number analyses using CyQUANT NF Cell Proliferation Assay. Data are shown as metabolic activity per million cells, depicted as box plots (n = 4). Data distribution and significance were calculated with Bonferroni test and two-way ANOVA. Medium-dependent differences: ** *p* < 0.01, *** *p* < 0.001.

Since the expansion and long-term culture of osteoblasts in vitro is rather hampered by calcium phosphate deposition and mineralization, we have been using calcium depleted medium in our cell culture for several years. However, a calcium-free environment is far from the in vivo situation in human bone. In order to better mimic the in vivo situation and to determine differential effects of calcium-depleted and calcium-containing medium conditions on activity of ion-exposed human osteoblasts, the standard osteogenic cell culture medium was compared with standard Dulbecco’s modified Eagle medium (Figure 3). The main differences between both media are the glucose concentration as well as the calcium and glutamine content. While the osteogenic medium is characterized by a low glucose concentration (1 g/L as well as calcium and glutamine depletion, DMEM contains high glucose (4.5 g/L), calcium chloride (0.26 g/L) and L-alanyl-L-glutamine (0.86 g/L). Since the respective bivalent ions Co(2+) or Ni(2+) would compete with Ca(2+) in the cell culture medium, our main hypothesis was that cellular activity was less affected in Ca-enriched medium after treatment with bivalent metal salts. We assumed that increased calcium levels in the cell culture allow osteoblasts to take up Ca(2+) rather than Co(2+) or Ni(2+), which may affect cell activity positively. With regard to our results, similar values of metabolic activity were detectable for untreated cells and ion-exposed cells in concentrations between 10 µM and 100 µM for Co(2+) and Ni(2+). At the highest ion concentration of 500 µM, a significant difference (Co: *p* = 0.0061; Ni: *p* = 0.004) between both media was determined with lower cell activity rates for Ca(2+) depleted medium. In Cr(3+)-exposed osteoblast, no differences between both media were detectable.

### 3.3. Expression of TLR4

**Figure 4:**
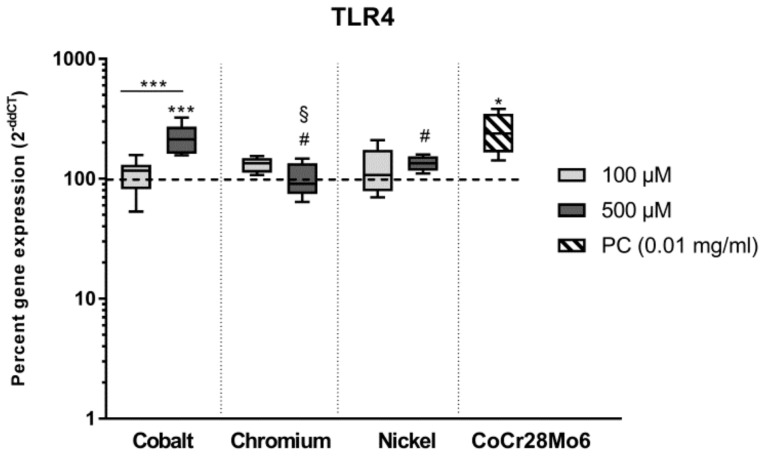
Relative transcript abundance of *TLR4* following exposure to metal salts. Gene expression levels were determined via semiquantitative real-time PCR in human osteoblasts treated with the respective metal salt concentration or particles (PC). Gene expression results are depicted as median and minimum/maximum values (n = 6) of the percentage of 2^(−ΔΔCt)^ related to the untreated control (100%). Significances between groups were calculated with RM two-way ANOVA using ΔCT values. Post hoc testing was performed with Tukey’s multiple comparison test: * *p* < 0.05, *** *p* < 0.001; differences to cobalt: ^#^ *p* < 0.05; differences to nickel: ^§^ *p* < 0.05.

Downstream processes are initiated by implant debris recognition via toll-like receptor (TLR) 4 known as a relevant receptor in aseptic implant loosening [30]. To determine *TLR4* gene expression in osteoblasts, cells were treated with 100 µM and 500 µM metal salt solutions since cell activity studies indicated significant differences between these concentrations. RM two-way ANOVA revealed significant differences for “type of metal salt” (*p* = 0.0132) and “concentration” (*p* = 0.0224) with a strong interaction between both factors (*p* < 0.0001). This was confirmed by post hoc testing (Figure 4). The positive control (CoCr28Mo6) as well as Co ions at the concentration of 500 µM, but not at 100 µM, significantly induced *TLR4* gene expression compared to the untreated control (*p* = 0.0239 and *p* < 0.0001, respectively) after 48 h. Co ions at 500 µM significantly enhanced *TLR4* transcripts compared to Cr ions (*p* < 0.0001) and Ni ions (*p* = 0.0006) at the same concentration. Meanwhile, Ni(2+) at 500 µM also showed an upregulation of mRNA when compared to Cr(3+) at 500 µM (*p* = 0.0122)—this effect did not reach significance when compared to the untreated control (*p* = 0.1749). 

### 3.4. Osteogenic Differentiation and Bone Remodeling after Exposure to Co-, Cr- and Ni-Ions

**Figure 5:**
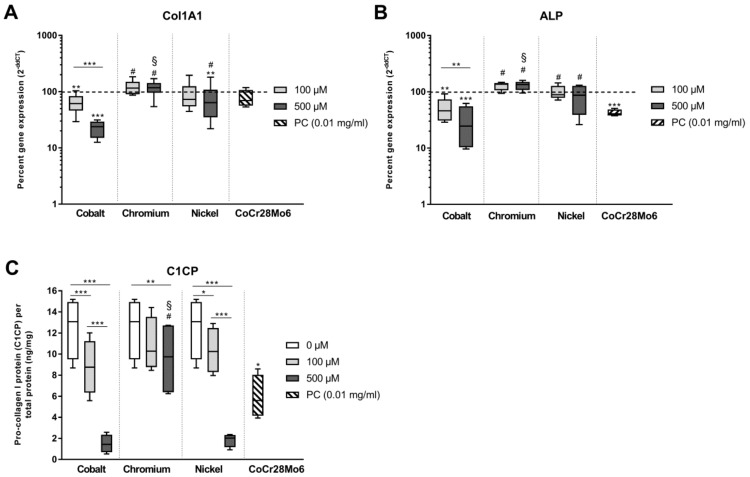
Relative transcript abundance of osteoblastic differentiation markers following exposure to metal salts. Gene expression levels of *Col1A1* and *ALP* by q-PCR (**A**,**B**) were determined in human osteoblasts treated with the respective metal salt concentration or particles (PC). Gene expression results are depicted as median and minimum/maximum values (n = 6) of the percentage of 2^(−ΔΔCt)^ related to the untreated control (100%). (**C**) Cell culture supernatants were used to evaluate the concentration of released pro-collagen 1 protein (C1CP per total protein content) by ELISA. Data are shown as median and minimum/maximum values (n = 4). Significances between groups were calculated with RM two-way ANOVA using ΔCT values for gene expression and values of the specific protein amount normalized to total protein content for protein expression. Post hoc testing was performed with Tukey’s multiple comparison test: * *p* < 0.05, ** *p* < 0.01, *** *p* < 0.001; differences to cobalt: ^#^ *p* < 0.05; differences to nickel: ^§^ *p* < 0.05.

The osteogenic differentiation capacity of human osteoblasts was analyzed after treatment with metal salts (100 µM and 500 µM). Collagen type 1, the main component of bone tissue, was clearly affected in human osteoblasts by bivalent ions (Figure 5, RM two-way ANOVA for gene expression: “type of metal salt” *p* = 0.0002, “concentration” *p* = 0.0024 and interaction of both *p* < 0.0001 [5A] and RM two-way ANOVA for protein synthesis: “type of metal salt” *p* = 0.0020, “concentration” *p* = 0.0002 and interaction of both *p* < 0.0001 [5C]). In detail, Co(2+) reduced both transcripts as well as protein levels in a concentration-dependent manner. Similar results were observed after exposure to Ni(2+), however, especially for gene expression, the reduction was not as pronounced as for Co ions. Exposure to 500 µM Co ions resulted in significantly lower mRNA transcripts than after exposure to Ni ions (*p* < 0.001). These results were comparable to *Col1A1* mRNA and protein expression rates of particle-treated human osteoblasts (positive control). Treatment with Cr(3+) did not affect transcript abundance and only slightly reduced protein levels (*p* = 0.0054 to untreated control). Besides collagen type 1, alkaline phosphatase (ALP) as another osteogenic differentiation marker was analyzed on transcript level and also showed significant variations with regard to the two tested variables (RM two-way ANOVA “type of metal salt” *p* < 0.0001, “concentration” *p* = 0.0143, and interaction of both *p* < 0.0001 [5B]). CoCr28Mo6 particles and Co ions significantly reduced *ALP* mRNA in osteoblasts after two days of exposure (*p* = 0.0010, *p* = 0.0014 and *p* < 0.0001 for particles, 100 µM and 500 µM to untreated control, respectively). The effect of Co(2+) was clearly concentration-dependent. Neither Ni nor Cr salts showed a significant influence on *ALP* gene expression and therefore differed significantly from Co at both tested concentrations (Cr to Co: *p* < 0.0001 at 100 and 500 µM; Ni to Co: *p* = 0.0003 and *p* < 0.0001 at 100 and 500 µM, respectively). 

**Figure 6:**
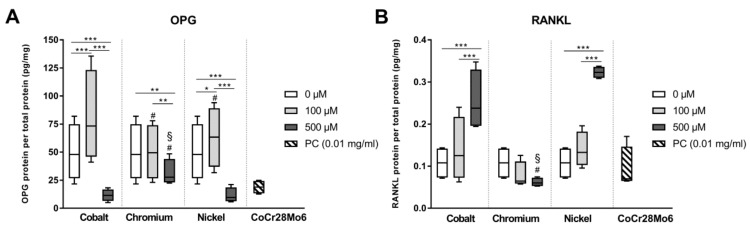
Protein expression of markers for bone remodeling following exposure to metal salts. Cell culture supernatants were used to evaluate the concentrations of released osteoprotegerin (OPG, **A**) and receptor activator of nuclear factor κb ligand (RANKL, **B**) by LEGENDplex™. Data are shown as median and minimum/maximum values (n = 4). Significances between groups were calculated with RM two-way ANOVA using values of the specific protein amount normalized to total protein content. Post hoc testing was performed with Tukey’s multiple comparison test: * *p* < 0.05, ** *p* < 0.01, and *** *p* < 0.001; differences to cobalt: ^#^ *p* < 0.05; differences to nickel: ^§^ *p* < 0.05.

OPG is released by human osteoblasts to counteract activity of RANKL which directly influence osteoclastogenesis. Both bone remodeling markers are synthesized by human osteoblasts. *RANKL* and *OPG* mRNA were neither detected in treated nor in untreated osteoblasts as Ct values were higher than the threshold of 28 cycles. However, on protein level, both bone remodeling marker were detectable. Treatment with Co(2+) and Ni(2+) at 500 µM led to a reduced soluble OPG protein content in the cell culture supernatants (Figure 6A) while RANKL protein was clearly upregulated (Figure 6B). Exposure to Cr(3+) reduced both, OPG and RANKL protein biosynthesis, in human osteoblasts. However, the effect was only significant for OPG synthesis and seems rather due to the generally reduced cellular activity (see Section 3.1) after incubation with Cr ions. The observed reduction of OPG synthesis by CoCr28Mo6 particles did not reach significance (*p* = 0.0568) and RANKL protein synthesis was not affected by particles. It is noteworthy that despite the clearly counteracting trends on OPG and RANKL synthesis by Co and Ni ions the amount of OPG released by osteoblasts still far exceeded the amount of RANKL produced (median values ranging from 9.54 to 73.30 pg/mg total protein for OPG and from 0.06 to 0.32 pg/mg total protein for RANKL).

### 3.5. Induction of Inflammation

**Figure 7:**
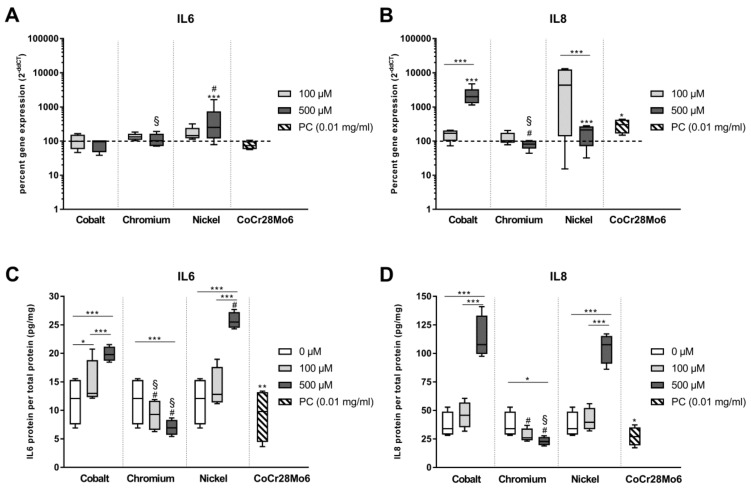
Expression of inflammatory mediators following exposure to metal salts. Gene expression levels of *IL6* and *IL8* by q-PCR (**A**,**B**) were determined in human osteoblasts treated with the respective metal salt concentration or particles (PC). Gene expression results are depicted as median and minimum/maximum values (n = 6) of the percentage of 2^(−ΔΔCt)^ related to the untreated control (100%). Cell culture supernatants were used to evaluate the concentration of released IL6 and IL8 protein (per total protein content) by ELISA (**C**,**D**). Protein data are shown as median and minimum/maximum values (n = 4). Significances between groups were calculated with RM two-way ANOVA using ΔCT values for gene expression and values of the specific protein amount normalized to total protein content for protein expression. Post hoc testing was performed with Tukey’s multiple comparisons test: * *p* < 0.05, ** *p* < 0.01, *** *p* < 0.001; differences to cobalt: ^#^ *p* < 0.05; differences to nickel: ^§^ *p* < 0.05.

In our previous in vitro studies, we could show that IL6 and IL8 are the most relevant pro-inflammatory mediators released by human osteoblasts in response to wear and corrosion products. In this study, *IL6* and *IL8* mRNA and protein expression levels were evaluated after exposure to Co(2+), Ni(2+) and Cr(3+) (Figure 7). When analyzing the gene expression profiles, *IL6* mRNA expression was only induced by Ni(2+) at 500 µM (*p* = 0.0006) while *IL8* mRNA expression levels were elevated by exposure to 500 µM Co, 500 µM Ni and to CoCr28Mo6 particles (*p* < 0.0001, *p* < 0.0001 and *p* = 0.0276, respectively) compared to the untreated control. The release of IL6 into the cell culture supernatant seemed independent from an induction of the gene expression as significant, concentration-dependent increases of IL6 and IL8 protein were determined in the cell culture supernatants after exposure to the bivalent ions Co and Ni. Interestingly, CoCr28Mo6 particles, which were used as a positive control, significantly reduced IL6 and IL8 release into the supernatant after 48 h compared to the untreated control (IL6: *p* = 0.0031, IL8: *p* = 0.0197). Again, incubation with Cr(3+) led to reduced protein levels of IL6 and IL8 in the supernatants, which is in accordance with the observation of a reduced cellular activity and lower protein synthesis for OPG and RANKL as reported in Section 3.1 and Section 3.4. In general, higher amounts of IL8 were released by osteoblasts than for IL6 with a maximum of 27.68 pg/mg total protein for IL6 and a maximum of 141.00 pg/mg total protein for IL8. 

## 4. Discussion

The corrosion of metal implants can lead to the release of free ions, which affect cellular behavior either locally or systemically. In this study, we analyzed the effects of different Co and Cr ions on osteoblastic survival, metabolism, and inflammation potential and compared the results with CoCr28Mo6 particle-exposed cells in order to assess the grade of toxicity. Since Ni is a common component in metal alloys, we additionally treated cells with Ni ions. The main purpose here was to compare the effects of different bivalent ions (Ni and Co) on the above mentioned cell processes.

For the experiments, we used Co(2+), Cr(3+) and Ni(2+) ions derived from metal salts. The tested concentrations were similar to those found in periprosthetic soft tissue [18] but lower than those reported for mineralized bone tissue [22]. The authors explained the relative high values in the mineralized bone tissue with the fact that the tissue was derived from the immediate vicinity, i.e., within a few millimeters, of the implant. This already highlights the difficulty to determine the relevant concentrations for in vitro-testing as these might differ depending on the distance from the implant but also depend on the conditions that aid corrosion. Also, when analyzing the in vivo concentrations, there is no differentiation whether the metals occur in form of ions or particles. Thus, our tested concentrations can only represent a rough estimate of the in vivo situation. Within the applied concentration range we were able to show that Co(2+) had a concentration-dependent influence on cell activity. Although the cell number remained (Figure S1) almost stable, an increase of metabolic activity (Figure S2) was initially detectable between 10 µM and 100 µM ion treatment. This effect can be explained by the fact that Co(2+) is able to surmount the cell membrane via membrane transport systems similar to Ca(2+). Intracellular, cobalt can then trigger hypoxia-like reactions in the cell, which lead to the upregulation of HIF-associated genes resulting in enhanced glycolytic reactions [31]. However, the highest Co(2+) concentration (500 µM) seemed to be more cytotoxic, not least because of a decline in cell number (Figure S1). The cytotoxicity of Co(2+) has been associated with enhanced oxidative stress, the formation of ROS, and DNA damage [2,32]. Consequentially, apoptosis or necrosis occurs [2,32] which was also detectable in our previous study [21]. Additionally, the toxic effects might be further explained by the inhibition of Ca(2+) entry and Ca(2+) signaling, leading to the intracellular competition of Co(2+) and Ca(2+) with intracellular Ca(2+) binding proteins affecting cellular behavior [6]. The results of our comparative analysis of Ca-containing and –depleted media might reflect the effect described by Scharf et al. (2014) [6]. Osteoblasts in Ca-containing medium showed increased metabolic activity compared to cells incubated in calcium-depleted medium after exposure to 500 µM Co(2+). We assume that the increased glucose content of the calcium-containing medium did not cause the increased metabolic activity, since no differences in cell activity were detected both in untreated cells and at the lower Co(2+) concentrations. As Co(2+) cannot be actively transported out of the cell, intracellular accumulation and cytotoxicity occurs [31]. Thus, in consideration of our results we rather hypothesize that, despite the increased presence of Co(2+), the cells prefer the Ca influx in Ca-enriched medium, resulting in a reduced intracellular Co(2+) accumulation, fewer cytotoxic effects and restored/increased metabolic activity. Bivalent Ni ions might affect osteoblasts via the same mechanism, but are however more cytotoxic than Co(2+). This was reflected by our observation of reduced cell numbers, especially at 500 µM and a loss of the actin filament structure.

In comparison to bivalent metal ions, the cell number was not affected by Cr(3+). However, a decrease in cell metabolism was detectable without concentration-dependent differences. This effect can be explained by the fact that Cr(3+) ions cannot enter the cell membrane [33] and therefore accumulate at the cell membrane [34], which may have an effect on cell metabolism. On the other hand, Magone et al. (2015) described the possibility of Cr(3+) oxidation to Cr(4+) in order to enter the cell membrane [7]. Intracellular, Cr(4+) conjugates with proteins resulting in reduction of Cr(4+) to Cr(3+) [7,32]. Although Shrivastava et al. (2002) mentioned that cell degeneration, DNA damage, and ROS accumulation is assigned to be the result of Cr(4+) exposure [23], other studies have revealed a release of ROS in the presence of Cr(3+) not least because of the rapid Cr (4+) reduction [6,7,32]. Additionally, cellular damage and the induction of apoptotic pathways by Cr(3+) were also described by Rudolf and Cervinka (2009) [35]. 

Exposure to CoCr28Mo6 particles resulted in unaffected cell activity compared to untreated cells. Compared to ion exposure, particle treatment led to an activity level similar to 100 µM Co(2+) or to obviously enhanced levels compared to Cr(3+). One simple explanation for this result can be the differing mechanism for the uptake and intracellular availability of ions and particles which directly affect cellular activity. As mentioned before, ions can enter the cell membrane via different ion-mediated transporter systems while particles have to be recognized by receptors or actively incorporated via phagocytosis [29,32]. On the other hand, the corrosion of particles within the cell culture media, or by cell-mediated mechanisms including phagocytosis, cannot be excluded. Thus, the released ions from the particles would have the same effect as Co(2+) ions from the metal salts on triggering the previously mentioned hypoxia-like reactions. However, the corrosion-mediated release of ions in the cell culture medium has not been examined yet, but will be the subject of further investigations.

A limitation for the determination of metabolic activity via WST-1 might be that this assay is influenced by the presence of superoxide, which is associated with phagocytosis and oxidative burst [36]. Therefore, the results for metabolic activity can also reflect the presence of ROS, as indeed WST-1 was used for the measurement of superoxide production [36]. However, WST-1 was performed in ion-free media, so we assumed that most of the results were caused by the reduction of formazan via the mitochondrial respiratory chain. Future experiments will establish whether the changes observed in metabolic activity after particle and metal ion exposure are linked to ROS production in our cells.

Intracellular downstream processes, including ROS production and oxidative burst, can be induced by a pathogen or substance binding on surface receptors. Here, toll-like receptors (TLR) recognize a wide spectrum of exogenous and endogenous danger signals [30] and therefore play a central role in the induction of intracellular cascades, especially in macrophages. Since TLR 4 is known to be the most relevant receptor in aseptic implant loosening [30,37], we looked at TLR4 sensitization in human osteoblasts after exposure to metal salts and controls. We initially assumed that CoCr28Mo6 particles primarily induce TLR4 expression and further downstream processes [38] while ions enter the cell membrane via channels and initiate immunological reactions. However, our results indicated that metal ions, especially Co(2+), have almost the same impact on *TLR4* gene expression as particles. It seems likely that the increased release of IL6 and IL8 can be directly linked to TLR4 activation. This is also supported by *TLR4* and IL6/IL8 results of Cr(3+) and Ni(2+) exposed cells. Lawrence and co-workers already showed, that Co(2+) activates TLR 4 which is in turn associated with enhanced chemokine release [39,40]. In contrast, Samelko et al. (2016) reported that CoCr particles did not preferentially activate TLR4 induced inflammation [41]. This is supported by our previous [28] as well as our current findings of reduced IL6 and IL8 secretion after particle exposure. Here, the release of pro-osteolytic mediators might be induced by other downstream mechanism such as inflammasome activation [41,42]. However, an impact of metal ions on inflammasome activation cannot be excluded as a strong link between ion channel expression and inflammasome activation has been described [43]. Thus, to finally prove the link between our findings of ion-mediated IL6 and IL8 release and TLR4 activation, further studies have been carried out, especially for the quantification of TLR4 on the surface of osteoblastic cells. 

Osteoblasts exposed to metal ions and CoCr28Mo6 particles showed reduced capacity of differentiation which was almost in accordance to our previous work [21,28]. Interestingly, Drynda et al. (2018) reported in their study that Cr(3+) instead of Co(2+) led to a concentration-dependent inhibition (10 μM to 250 μM) of osteoblastic mineralization [22]. This fact could not be proven in our work, since we found significantly reduced osteogenic differentiation at higher Co(2+) concentration, whereas Cr(3+) had significantly less (Col1 protein) or no effects (*Col1A1, ALP*). Our present data show that not only CoCr28Mo6 particles affect extracellular matrix production and hence bone formation processes, but additionally the presence of bivalent metal ions can inhibit collagen type 1 synthesis. Bone degradation and osteolysis can be further advanced by altered OPG/RANKL ratios as these affect osteoclastogenesis and bone resorption. Here, it should be noted that OPG prevents bone loss while RANKL promotes osteoclastic resorption [7]. Our data indicated that mainly bivalent ions have a significant impact on OPG/RANKL ratios. Although OPG protein concentrations were clearly elevated compared to RANKL, concentration-dependent effects were found: while OPG was significantly downregulated at 500 µM, RANKL protein was significantly upregulated. As a result, OPG/RANKL ratio at 500 μM was only about one tenth of the ratio at 100 μM. This was in contrast to the study of Zijlstra et al. (2012) who revealed a time-dependent effect of Co and Cr ions on *OPG/RANKL* mRNA ratio without concentration-dependent differences [44]. However, they only calculated ratios via gene expression results and not on protein data, which is important to demonstrate osteolytic reactions in the periprosthetic tissue. In our study, we were not able to detect *OPG* and *RANKL* transcripts although we used clearly lower metal ion concentrations. Nonetheless, our protein data can provide important information about the effects of ion exposure on bone remodeling. In this context, co-cultures should be carried out in the future to prove the effects of osteoclastogenesis and further bone resorption as already shown in our previous study [21]. 

## 5. Conclusions

Our data show that especially Co(2+) as a bivalent ion has a significant influence on osteoblastic activity, differentiation, and inflammatory processes. In this work, we were further able to show clear differences between Co(2+) and Cr(3+), and thus to assess the toxicity of both ions. We were able to demonstrate an increased sensitization of human osteoblasts resulting in unbalanced bone remodeling as shown by lower collagen type production, decreased OPG/RANKL ratio, as well as the induction of inflammation. Moreover, corrosion products induced cellular downstream processes faster, i.e., after a short-time exposure of only 48 h, compared to CoCr28Mo6 particles where significant effects were shown after 96 h [28]. 

## Figures and Tables

**Table 1:** Overview of primer sequences for qRT-PCR.

Primer	Forward (5′-3′)	Reverse (5′-3′)
Alkaline phosphatase (ALP)	cattgtgaccaccacgagag	ccatgatcacgtcaatgtcc
Collagen 1 (Col1A1)	acgaagacatcccaccaatc	agatcacgtcatcgcacaac
Hypoxanthine guanine phosphoribosyltransferase (HPRT)	ccctggcgtcgtgattagtg	tcgagcaagacgttcagtcc
Interleukin 6 (IL-6)	tggattcaatgaggagacttgcc	ctggcatttgtggttgggtc
Interleukin 8 (IL-8)	tctgtgtgaaggtgcagttttg	atttctgtgttggcgcagtg
Toll-like receptor 4 (TLR 4)	ggtcagacggtgatagcgag	tttacgggccaagtctccacg

## Supplementary Materials

The following are available online at https://www.mdpi.com/1996-1944/12/17/2771/s1, Figure S1: Cell numbers of human osteoblasts after exposure to metal salts. Untreated cells served as controls (0 µM). Osteoblasts were treated with different concentrations of Co(2+), Cr(3+) and Ni(2+) over 48 h. Afterwards cell number was determined via CyQUANT NF Cell Proliferation Assay. Data are depicted as box plots (n = 7). Significance was calculated with concentration-dependent differences: * *p* < 0.05, ** *p* < 0.01, *** *p* < 0.001; differences to cobalt: ^#^ *p* < 0.05; differences to nickel: ^§^ *p* < 0.05, Figure S2: Metabolic activity of human osteoblasts after exposure to metal salts. Untreated cells served as controls (0 µM). Osteoblasts were treated with different concentrations of Co(2+), Cr(3+) and Ni(2+) over 48 h. Afterwards metabolic activity was determined via water soluble tetrazolium salt (WST-1) assay. Data are depicted as box plots (n = 7). Significance was calculated with concentration-dependent differences: * *p* < 0.05, *** *p* < 0.001; differences to cobalt: ^#^ *p* < 0.05; differences to nickel: ^§^ *p* < 0.05.
