# Supplementary material for: Analysis of Cellular Activity and Induction of Inflammation in Response to Short-Term Exposure to Cobalt and Chromium Ions in Mature Human Osteoblasts
Source: Materials (Basel). 2019 Aug 28;12(17):2771. doi: 10.3390/ma12172771 (PMC6747798; doi:10.3390/ma12172771)
Supplement: Supplementary file 1 [file materials-12-02771-s001.pdf]

*materials*

*Article*

# Analysis of Cellular Activity and Induction of Inflammation in Response to Short-Term Exposure to Cobalt and Chromium Ions in Mature Human Osteoblasts

**Anika Jonitz-Heincke [1,*], Marie-Luise Sellin [1], Anika Seyfarth [1], Kirsten Peters [2], Brigitte Mueller-Hilke [3], Tomas Fiedler [4], Rainer Bader [1] and Annett Klinder [1]**

[1] Biomechanics and Implant Technology Research Laboratory, Department of Orthopedics, Rostock University Medical Centre, Doberaner Strasse 142, Rostock 18057, Germany
[2] Department of Cell Biology, Rostock University Medical Center, Schillingallee 69, Rostock 18057, Germany
[3] Institute for Immunology, Rostock University Medical Center, Schillingallee 70, Rostock 18057, Germany
[4] Institute for Medical Microbiology, Virology and Hygiene, Rostock University Medical Center, Schillingallee 70, Rostock 18057, Germany
* Correspondence: anika.jonitz-heincke@med.uni-rostock.de; Tel.: +49-381-494-9306

## Supplementary Figures

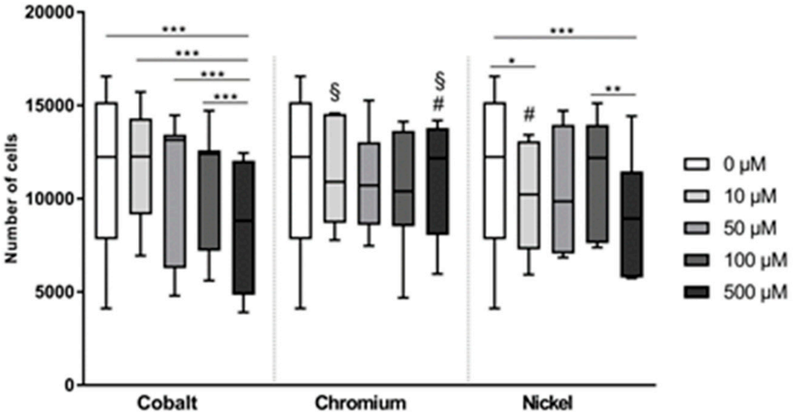


**Figure S1:** Cell numbers of human osteoblasts after exposure to metal salts. Untreated cells served as controls (0 µM). Osteoblasts were treated with different concentrations of Co(2+), Cr(3+) and Ni(2+) over 48 h. Afterwards cell number was determined via CyQUANT NF Cell Proliferation Assay. Data are depicted as box plots (n = 7). Significance was calculated with concentration-dependent differences: *$p < 0.05$, **$p < 0.01$, ***$p < 0.001$; differences to cobalt: $^{\#}p < 0.05$; differences to nickel: $^{\S}p < 0.05$.

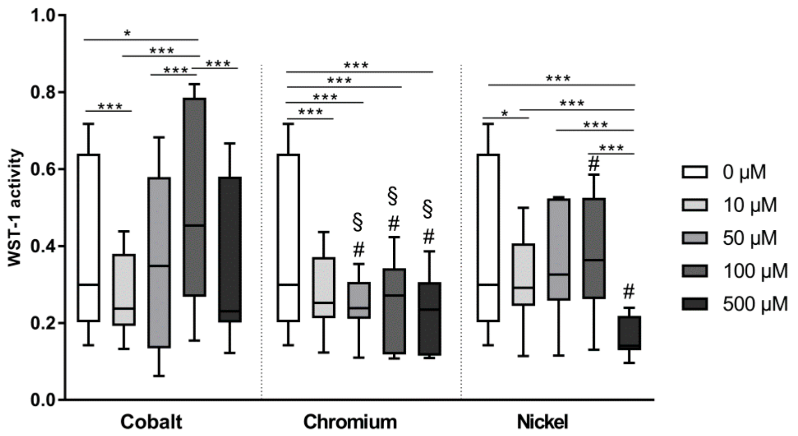


**Figure S2:** Metabolic activity of human osteoblasts after exposure to metal salts. Untreated cells served as controls (0 μM). Osteoblasts were treated with different concentrations of Co(2+), Cr(3+) and Ni(2+) over 48 h. Afterwards metabolic activity was determined via water soluble tetrazolium salt (WST-1) assay. Data are depicted as box plots (n = 7). Significance was calculated with concentration-dependent differences: *$p < 0.05$, ***$p < 0.001$; differences to cobalt: $^{\#}p < 0.05$; differences to nickel: $^{\S}p < 0.05$.
